# Differences in Rates of Low Birth Weight among Prefectures in Japan: An Ecological Study Using Government Statistics Data

**DOI:** 10.3390/children9030305

**Published:** 2022-02-23

**Authors:** Tasuku Okui, Naoki Nakashima

**Affiliations:** Medical Information Center, Kyushu University Hospital, Fukuoka 812-8582, Japan; nakashima.naoki.351@m.kyushu-u.ac.jp

**Keywords:** low birth weight, Japan, body mass index, vital statistics

## Abstract

The differences in the rates and trends of the overall low birth weight and term low birth weight in recent years are unknown for the Japanese prefectures. In this ecological study, we revealed the rates for each prefecture and investigated the factors affecting the regional differences in these outcomes. Aggregated vital statistics data from 2007 to 2019 were obtained from the Ministry of Health, Labour, and Welfare in Japan. The association between the outcomes and the variables, including the infants’ birth characteristics, medical characteristics, and socioeconomic characteristics of the prefectures, were analyzed. An analysis of repeated-measures data was conducted using the data from 2013 and 2018 for each prefecture. The trend for the rates of overall low birth weight and term low birth weight over the years differed among the prefectures. Moreover, the proportions of multiple births and lean (body mass index <18.5 kg/m^2^) and obese (body mass index ≥25.0 kg/m^2^) women had a statistically significant positive association with both the overall low birth weight rate and the term low birth weight rate among the prefectures. It was suggested that to resolve the difference in these outcomes among the prefectures, being obese or underweight needs to be addressed in mothers.

## 1. Introduction

The rates of low birth weight are representative of perinatal health outcomes worldwide and are one of the indicators of maternal and child health. Low birth weight is associated with preterm birth and intrauterine growth retardation. Low birth weight infants have a higher risk of neonatal and infant mortality or cardiovascular diseases [1,2,3,4]; thus, preventing these conditions is an important issue for public health and social security in Japan. There is a disparity in the outcome among countries, and the low birth rate in Japan is lower than the estimated worldwide prevalence [5,6]. On the other hand, the low birth rate showed an increase from the late 1970s to the 2010s [7], and changes in body mass index (BMI), maternal aging, and an increase in cesarean sections are believed to be contributing factors. Although some studies have investigated an association between maternal characteristics and birth weight trends in Japan [7,8,9], few studies have investigated an association between the outcome and multiple types of factors, such as sociodemographic or physical characteristics, using nationwide statistics data in Japan.

Regional differences in the rates of preterm birth or low birth weight within a country have been investigated in other countries, and a disparity in the outcomes has been observed [10,11,12,13]. There are regional differences in the rates of infants with low birth weight among prefectures in Japan [14,15,16]; however, no studies have investigated the regional differences in the term low birth weight among Japanese prefectures. The term low birth weight is defined as low birth weight excluding preterm birth [5,17,18,19], and is used as an indicator of fetal growth restriction. In Japan, a decreasing trend in the rate of the term low birth weight was observed in recent years [5], although there might exist some differences in the trends among regions. In addition, to the best of our knowledge, no studies have been conducted assessing the differences in low birth weight rates among the Japanese prefectures in recent years. Moreover, identifying factors affecting the regional differences is important in understanding the reasons for these differences. Low birth weight is known to be affected by the socioeconomic or demographic characteristics of regions in other countries [10,12], and there is a possibility that regional differences in these characteristics also affect the outcome also in Japan.

In this study, we assessed the rate of low birth weight in prefectures in Japan and investigated the factors affecting these differences by an ecological study using all of the birth data in the vital statistics.

## 2. Materials and Methods

Vital statistics data for births were used for the analysis. We requested the aggregated data from the Ministry of Health, Labour, and Welfare by a made-to-order aggregation based on the Statistics Act (Heisei 19 nen, Article 53). Data on the number of births by maternal age group, year, prefecture, birth weight, and gestational age were obtained. Data from 2007 to 2019 on all 47 prefectures in Japan were obtained. In this study, we used the operational definitions for the two outcomes, i.e., low birth weight rate and the term low birth weight. Low birth weight was defined as infants whose birth weight was less than 2500 g [7,9]. Term birth was defined as births at 37 weeks of gestation or later, and infants with term low birth weight were defined as those whose birth weight was less than 2500 g among the term births, similar to previous studies [17,18]. The cases with missing data regarding gestational age, birth weight, or maternal age were excluded from the analysis.

To investigate an association between the outcomes and predictors via an ecological study using prefectural data, we used variables related to the medical characteristics, birth characteristics, maternal characteristics, and socioeconomic factors of the prefectures: the proportion of multiple births (%); the proportion of live births from mothers aged under 20 years (%); the proportion of live births from mothers aged 40 years or more (%); the number of hospitals per 100,000 persons; the number of clinics per 100,000 persons; the number of hospitals with an obstetrics department per 100,000 persons; total population; population density; taxable income per capita (1000 yen); the proportion of female high school graduates going to a higher educational institution; the proportion of live births from unemployed households; the proportion of lean women (%); and the proportion of obese women (%) for each prefecture. Data on the number of multiple births and total births for each prefecture were obtained from the vital statistics [20]. We included hospitals with an obstetrics department or an obstetrics and gynecology department as hospitals with an obstetrics department, and the data on the number of hospitals and clinics were obtained from the Survey of Medical Institutions [21]. Taxable income for each prefecture was assessed in “the Survey on Taxation Status of Municipal Tax” by the Ministry of Internal Affairs and Communications [21], and area data of each prefecture were available from the Municipalities Area Statistics of Japan [21]. Data on the proportion of female high school graduates going to a higher educational institution were obtained from the School Basic Survey in Japan [21], and data on the proportion of live births from unemployed households were obtained from the vital statistics [20]. The numbers of lean and obese women for each prefecture were obtained from the National Database of Health Insurance Claims and Specific Health Checkups of Japan open data [22]. The Specific Health Checkups are conducted for insured persons and their dependents aged 40–74 years in all of Japan, and they are encouraged to receive a medical checkup concerning lifestyle-related diseases every year. Therefore, we used the Specific Health Checkups data only for women aged 40–49 years who are in the reproductive age groups. In contrast, we used all the birth data, regardless of the mother’s age, in the analysis. Data on the BMI classification for the recipients of the checkups in each prefecture are available, and the proportion of lean persons (BMI < 18.5 kg/m^2^) and of obese persons (BMI ≥ 25.0 kg/m^2^) was calculated. Although obesity is often judged based on the cut-off value of BMI ≥ 30.0 kg/m^2^ across the world [23,24], the cut-off value of BMI ≥ 25.0 kg/m^2^ is generally used in Japan and Korea [25,26,27,28,29]. Data on these explanatory variables in 2013–2018 were used for the analysis because all the data were publicly available only in these years.

For the statistical analysis, we calculated the overall low birth weight rate and term low birth weight rate for each prefecture by year. In addition, we calculated the overall low birth weight rate and term low birth weight rate in the analyzed periods for each prefecture and calculated the annual percent change (APC) of the outcomes for each prefecture. The APC was calculated by applying a linear regression model to the logarithm of the rate using year as an explanatory variable. Moreover, an ecological study was conducted to investigate an association between the outcomes and the characteristics of the prefecture. An ecological study is a type of epidemiological study that is conducted to investigate an association between a health outcome and risk factors using regional-level data as opposed to individual-level data, and it aims at identifying the risk factors of the health outcome by focusing on the differences in the regional characteristics. Therefore, regression analysis was performed using the data of each prefecture in this study. An analysis using the repeated-measures data of the prefectures was conducted using the data from 2013 and 2018 in each prefecture. A linear mixed effects model was used using the logarithm of the rate as an outcome, and each prefecture was treated as a random effect in the analysis. When regression analysis is used for data where multiple observations exist for a subject (often called panel data or repeated-measures data), a linear mixed model is often used. All the explanatory variables were scaled, and a standardized partial regression coefficient (SPRC) and its 95% confidence interval were calculated for each variable to compare their effects on the outcomes. The SPRC is a type of regression coefficient that is obtained when an explanatory variable is scaled before applying the regression model. By calculating the SPRC, we could compare the degree of association of an outcome variable among the explanatory variables. A statistically significant difference was set as <0.05, and all the statistical tests were 2-sided. All the statistical analyses were conducted using R version 3.6.3 (https://cran.r-project.org/bin/windows/base/old/3.6.3/, accessed on 23 February 2022).

## 3. Results

Table 1 shows the yearly number of births, low-birth-weight infants, number of term births, and term low-birth-weight infants in Japan. The number of births continuously decreased in these periods. In addition, the overall low birth weight rate and term low birth weight rate decreased from 2007 to 2019.

Table 2 shows the annual rate of low birth weight per 1000 births for each prefecture. The trend of the low birth weight rate in these periods differed depending on the prefecture. The rate for Okinawa was the highest among the prefectures in many of the years. In addition, the rates in Tochigi and Kagoshima were always above 100 in the analyzed periods.

Table 3 shows the annual rate of term low birth weight per 1000 births for each prefecture. The trend of the term low birth weight rate also differed depending on the prefecture. The values for prefectures such as Yamanashi, Kochi, and Okinawa tended to be large, whereas those for prefectures such as Ishikawa and Fukui tended to be small.

Table 4 shows the overall low birth weight rate and the term low birth weight rate per 1000 births in 2007–2019 for each prefecture and their APCs. The signs of APC of the overall low birth weight rate and the term low birth weight rate differed across prefectures.

Table 5 shows the median (Q1–Q3) for each characteristic in the prefectures used in the analysis. For the regression analysis, 282 (47 prefectures × 6 years) observations were used.

Table 6 shows results of the regression analysis. The proportion of live births from mothers aged 40 years or more had a statistically significant positive association with the rate of low birth weight at term. Hence, the higher the proportion of live births from mothers aged 40 or more years in a prefecture is, the higher the rate of term low birth weight of the prefecture tends to become. In addition, the proportion of multiple births and the proportions of lean (body mass index (BMI) < 18.5 kg/m^2^) and obese women (BMI ≥ 25 kg/m^2^) had a statistically significant positive association with both the overall low birth weight rate and the term low birth weight rate. Therefore, the proportions of lean and obese women in a prefecture were positively associated with the prefectural low birth weight rate. In addition, the absolute value of SPRC for the proportion of obese women was the largest among the explanatory variables, and the proportion of obese women was suggested to be the largest factor related to the regional differences in low birth weight.

## 4. Discussion

In this ecological study, we aimed to reveal the low birth weight rates in different prefectures in Japan and identify the factors that determine the differences in these rates. As a result, we revealed a trend in the low birth weight rate for each prefecture in Japan and identified some factors associated with these regional differences. The proportion of multiple births were statistically significantly associated with low birth weight and term low birth weight, and the proportion of births by women with advanced age was statistically significantly associated with the term low birth weight rate. Multiple births are a well-known factor contributing to preterm birth and low birth weight [30]. In addition, older maternal age is known to be a risk factor for intrauterine growth restriction [31,32].

As shown in the results, the number of births in Japan is rapidly decreasing over the years. Tendencies to not marry and marry late are directly related to the decline in fertility in Japan, and factors such as the increased burden of parenting, women’s empowerment, and an increase in the rate of irregular employment are believed to be the possible causes [33]. In addition, the overall low birth weight rate and the term low birth weight rate showed a decreasing trend in Japan, and regression analysis indicated that the year was negatively associated with these outcomes. Although the reason for the phenomenon remains to be uncovered, an increase in the rate of participation in prenatal care is one possible reason. From 2009, public subsidies for prenatal care largely increased in Japan, and it was shown in a prefecture in Japan that this policy increased the number of prenatal care visits and decreased the overall low birth weight rate [34]. An association between low birth weight and prenatal care visits has been previously reported [35,36], and an increase in the rate of participation in prenatal care is a possible reason for the decrease in the low birth weight rate. 

Pre-pregnancy obesity and underweight are both risk factors for low birth weight in infants [37,38]. In particular, an increase in the rate of underweight women is believed to be a main reason for the increase in the rate of low-birth-weight infants in recent years in Japan [9,39]. Poor nutritional intake and underweight in pregnant women led to poor maternal weight gain and affected optimal fetal growth [39]. On the other hand, the prevalence of obesity was more strongly associated with low birth weight in this study. Pre-pregnancy obesity is related to diabetes and hypertension, which are risk factors for low birth weight [40]. In addition, obese women have a higher risk of pregnancy complications, such as gestational hypertension and diabetes [41,42], and women with these pregnancy complications are at higher risk of preterm births and low-birth-weight infants [40,43]. There is also a possibility that rates of low birth weight in the past affected the obesity or underweight prevalence in the present. An association between underweight and low birth weight at birth has been shown among children in Japan [44], and lower birth weight was shown to be associated with underweight among children also in China [45]. In addition, one study found that low birth weight was associated with obesity in adults [46], while a meta-analysis showed that low-birth-weight infants have a decreased risk of later overweight [47]. A previous study investigating regional differences in low birth weight in Japan for 1975 and 1994 showed that the rates of low birth weight in Okinawa were also high among the prefectures in these periods [15], and it is possible that low birth weight affected physical characteristics in later years.

It has been suggested that regional differences in obesity and underweight possibly affect the regional differences in low birth weight among prefectures. Preventive measures for obesity are often promoted to prevent lifestyle-related diseases; however, obesity is also related to the birth weight of infants. Current differences in health statuses among prefectures can lead to differences in health statuses in subsequent generations. It has been suggested that lower maternal birth weight is associated with low-birth-weight infants in Japan [48]. Therefore, health guidance not only for middle-aged or older women with obesity but also for younger women with obesity might also be needed.

Disparities are known to exist in the rates of both infant and adult mortality among prefectures in Japan [49,50], and understanding and correcting the differences in perinatal outcomes among regions might contribute to improving these disparities in the future. There are some limitations in this study. First, this is an ecological study, and an ecological fallacy in the results is possible. Second, we could use only the data of 2013 and 2018 for the ecological analysis, and a further study using data from longer periods will be meaningful. Third, the BMI data from each prefecture were available for people aged 40–49 years because these age groups were participants in the Specific Health Checkups; we could not obtain the BMI data for those aged 15–39 years for each prefecture. In addition, participation in the Specific Health Checkups is not compulsory, and there is a possibility that people not participating in the checkups affected the differences among the prefectures. Fourth, other socioeconomic or environmental factors might also be related to the regional differences in the outcomes. For example, the percentage of pregnant women not participating in prenatal care is also considered to be related to the regional differences, and their data are not available. In addition, complications during pregnancy, such as gestational hypertension or diabetes, are considered to be associated with low birth weight; however, data on these characteristics were not publicly available. Moreover, we used made-to-order aggregation data from the Ministry of Health, Labour, and Welfare in Japan, and the variables that can be requested in one aggregation table were limited. We could not request other factors related to the outcomes, such as parity and household occupations at birth in the aggregation table.

## 5. Conclusions

We revealed regional differences in the rates of low birth weight and term low birth weight, and identified characteristics of prefectures associated with the regional differences by an ecological study using all of the birth data in Japan. As a result, we can see that trends in the rates differed depending on the prefectures in the analyzed periods. As a result of this ecological study, the proportion of live births from mothers aged 40 years or more had a statistically significant positive association with the rate of term low birth weight. In addition, the proportion of multiple births and the proportions of lean and obese women had a statistically significant positive association with both the low birth weight rate and term low birth weight rate.

## Figures and Tables

**Table 1:** Yearly number of births, low-birth-weight infants, number of term births, and term low-birth-weight infants in Japan.

Year	Number of Births	Number of Low-Birth-Weight Infants (%)	Number of Term Births	Number of Term Low-Birth-Weight Infants (%)
2007	1,089,196	105,090 (9.65)	1,026,116	61,910 (6.03)
2008	1,090,498	104,402 (9.57)	1,027,715	61,785 (6.01)
2009	1,069,486	102,610 (9.59)	1,008,522	60,970 (6.05)
2010	1,070,786	102,999 (9.62)	1,009,498	61,377 (6.08)
2011	1,050,300	100,328 (9.55)	990,046	59,383 (6.00)
2012	1,036,800	99,270 (9.57)	977,308	59,122 (6.05)
2013	1,029,459	98,579 (9.58)	970,245	58,477 (6.03)
2014	1,003,247	95,741 (9.54)	946,366	56,994 (6.02)
2015	1,005,367	95,174 (9.47)	949,265	57,155 (6.02)
2016	976,881	92,076 (9.43)	922,335	55,172 (5.98)
2017	945,840	89,328 (9.44)	892,315	53,177 (5.96)
2018	918,113	86,239 (9.39)	866,407	50,919 (5.88)
2019	864,978	81,435 (9.41)	816,457	48,403 (5.93)

**Table 2:** Annual rate of overall low birth weight per 1000 births for each prefecture.

	Year												
Prefecture	2007	2008	2009	2010	2011	2012	2013	2014	2015	2016	2017	2018	2019
Hokkaido	98.4	95.5	91.4	97.8	96.7	97.3	97.5	97.1	93.3	92.0	95.0	91.5	91.5
Aomori	96.1	94.5	92.2	95.0	94.4	95.0	93.4	86.9	87.4	84.5	88.5	99.2	95.3
Iwate	90.6	91.2	90.4	93.9	90.6	93.0	96.7	97.3	91.9	97.8	97.3	101.1	99.2
Miyagi	90.9	91.9	90.8	91.8	95.4	95.5	98.4	95.2	93.0	97.1	97.7	92.7	92.9
Akita	96.8	103.9	96.3	98.3	99.2	95.1	100.0	101.9	97.1	105.2	101.2	104.4	101.8
Yamagata	86.1	88.5	82.9	90.1	81.8	87.6	88.9	89.7	91.2	93.4	88.5	90.1	88.4
Fukushima	96.8	91.0	89.4	95.8	91.1	96.4	99.5	96.5	94.9	94.6	90.1	90.1	95.3
Ibaraki	96.7	95.5	90.3	95.8	94.5	97.5	97.1	94.9	96.9	93.7	93.1	94.5	95.2
Tochigi	100.5	101.3	104.9	103.1	105.3	104.8	109.5	102.9	105.2	104.2	105.1	105.7	101.5
Gunma	98.4	94.1	93.0	93.8	92.9	97.8	96.1	95.1	95.4	90.4	95.5	98.4	95.3
Saitama	94.7	94.5	94.2	95.3	93.9	95.2	96.4	94.3	94.6	91.6	94.1	94.0	95.0
Chiba	91.7	90.2	92.1	92.4	92.3	92.6	93.4	90.8	88.4	92.4	88.8	90.1	90.9
Tokyo	94.3	95.6	94.6	93.8	94.9	93.4	94.1	91.9	91.1	91.9	90.9	91.4	92.2
Kanagawa	96.3	97.3	96.1	96.2	95.5	97.1	93.5	95.4	94.5	94.8	95.7	93.2	94.6
Niigata	89.8	86.0	89.3	88.3	90.0	92.8	92.7	95.0	95.5	90.1	95.7	88.9	89.2
Toyama	97.3	85.1	101.2	88.8	86.9	88.9	81.5	86.2	88.5	97.4	88.2	88.4	87.1
Ishikawa	87.5	82.1	78.5	87.7	89.0	86.2	91.9	90.5	94.9	86.5	90.8	85.9	91.5
Fukui	88.6	93.2	85.5	84.7	82.3	88.2	86.2	81.8	87.0	87.2	82.1	86.7	89.0
Yamanashi	99.6	106.7	116.2	112.1	97.9	105.9	101.5	101.6	102.7	102.3	97.6	102.8	104.8
Nagano	95.8	94.9	101.0	96.9	99.9	92.7	95.0	95.0	93.8	95.6	92.8	90.6	95.8
Gifu	91.1	92.3	93.6	92.9	96.5	95.5	93.5	87.4	90.7	91.0	93.6	91.0	98.3
Shizuoka	103.5	101.9	103.2	101.6	101.2	101.6	102.2	99.9	100.6	101.2	98.2	98.9	97.1
Aichi	98.0	95.9	99.8	97.6	95.9	97.7	96.5	97.8	98.1	97.5	94.7	97.3	97.5
Mie	92.2	86.8	92.2	91.8	89.7	88.7	88.9	96.9	93.1	91.7	92.6	90.1	92.6
Shiga	94.0	93.8	92.6	94.2	98.7	96.4	93.5	98.3	92.6	91.6	94.4	92.2	90.1
Kyoto	99.0	99.0	98.3	100.6	97.8	98.2	97.1	96.5	94.6	96.2	95.1	94.2	94.1
Osaka	97.4	97.1	96.8	97.2	97.4	94.8	93.7	96.0	92.8	90.4	92.9	90.0	90.6
Hyogo	100.3	96.5	96.0	95.5	95.9	94.6	96.8	95.9	92.4	95.8	93.9	93.7	92.4
Nara	98.4	98.4	92.9	91.1	91.7	90.5	96.2	90.0	91.8	94.5	86.2	93.1	87.8
Wakayama	95.0	99.1	94.2	92.2	99.2	89.0	91.2	98.2	96.4	96.1	94.6	88.6	93.7
Tottori	88.0	89.8	89.0	98.8	99.2	93.6	99.0	102.3	96.3	100.1	104.2	100.5	100.8
Shimane	88.8	94.5	111.6	106.7	107.9	104.6	97.1	108.1	104.0	100.6	96.1	99.9	105.2
Okayama	89.1	85.8	93.9	87.7	87.9	90.9	90.2	92.7	91.6	86.5	91.2	90.2	79.2
Hiroshima	95.2	93.5	97.6	96.2	99.9	96.3	96.3	95.6	96.6	97.1	95.4	97.7	93.5
Yamaguchi	96.0	102.4	99.1	98.6	97.2	98.1	94.0	93.4	98.8	93.8	94.2	92.6	99.7
Tokushima	84.1	87.4	95.0	96.1	89.0	83.7	97.6	86.2	89.7	101.4	97.5	84.5	89.8
Kagawa	89.0	90.9	92.2	94.0	84.6	92.8	83.4	89.2	91.1	91.0	85.7	90.3	92.1
Ehime	91.9	90.6	92.0	90.9	93.8	86.4	89.6	88.0	94.0	86.5	92.0	94.5	86.8
Kochi	112.5	113.3	101.0	104.7	105.0	112.1	107.0	106.7	102.4	89.8	102.6	102.5	111.7
Fukuoka	104.2	101.7	100.1	102.0	98.3	98.6	99.5	97.4	99.1	96.4	97.5	97.2	94.6
Saga	96.0	96.4	89.8	98.1	91.0	90.9	97.2	94.3	91.3	93.7	97.3	89.1	92.6
Nagasaki	92.8	97.0	91.5	99.5	86.2	88.5	88.9	90.5	91.3	89.6	91.2	93.0	92.2
Kumamoto	100.4	98.9	97.6	92.9	91.8	96.6	89.3	94.6	94.9	87.3	93.4	89.6	91.0
Oita	92.8	89.4	94.0	95.1	88.5	91.1	92.8	86.0	94.6	96.5	95.2	95.9	100.6
Miyazaki	104.2	107.5	105.6	100.1	104.8	101.1	103.5	105.8	103.4	98.9	101.0	102.1	100.5
Kagoshima	100.4	104.3	106.4	104.2	105.3	101.8	104.3	111.5	104.5	103.1	114.9	107.0	107.9
Okinawa	117.9	109.5	115.3	111.9	106.3	115.9	113.8	114.5	109.1	112.7	110.8	109.9	111.5

**Table 3:** Annual rate of term low birth weight per 1000 births for each prefecture.

	Year												
Prefecture	2007	2008	2009	2010	2011	2012	2013	2014	2015	2016	2017	2018	2019
Hokkaido	60.1	58.2	55.8	60.7	57.0	58.5	58.1	58.6	57.0	55.6	56.4	56.4	56.4
Aomori	55.5	55.8	53.2	57.8	57.1	59.7	56.0	53.9	57.7	56.0	56.6	59.5	58.8
Iwate	54.8	53.9	53.2	58.3	57.2	59.0	61.7	57.8	55.4	58.3	59.3	60.5	57.0
Miyagi	53.6	54.9	53.6	54.0	57.0	56.3	57.0	55.5	54.8	59.7	57.3	55.5	54.0
Akita	56.7	63.6	58.1	57.8	59.6	58.3	56.7	62.8	63.3	69.0	67.0	64.6	64.2
Yamagata	52.3	55.3	50.1	54.2	51.9	54.9	52.6	57.2	58.0	61.0	56.4	50.5	54.9
Fukushima	58.5	57.6	54.5	63.6	59.3	61.9	64.4	61.6	61.5	60.4	59.9	57.0	61.3
Ibaraki	59.7	61.0	58.2	61.2	59.9	62.8	61.6	59.9	63.4	60.8	60.8	61.2	61.2
Tochigi	62.7	62.0	63.7	66.2	63.8	66.8	65.9	61.3	68.3	66.9	65.9	68.9	66.1
Gunma	64.5	59.9	60.5	59.9	60.1	63.4	60.9	60.0	61.2	56.7	62.2	62.7	60.1
Saitama	58.8	59.8	60.8	60.7	59.2	59.8	59.9	59.2	60.2	57.7	59.6	58.3	58.9
Chiba	57.7	57.6	57.0	58.9	57.4	57.6	59.8	57.9	55.1	58.2	56.7	57.0	57.4
Tokyo	60.4	60.5	60.0	60.7	59.9	59.4	60.9	58.5	58.5	59.2	58.1	58.1	59.0
Kanagawa	62.7	63.8	62.0	61.7	61.1	62.7	61.0	61.7	62.6	60.8	60.9	57.5	59.2
Niigata	56.6	53.0	54.7	53.9	54.0	58.2	52.5	58.4	58.7	56.8	60.6	53.7	56.2
Toyama	58.6	47.2	61.0	55.4	56.6	55.1	49.1	56.0	58.7	58.8	56.1	57.3	56.2
Ishikawa	54.0	49.1	47.1	54.3	55.8	53.9	57.5	58.6	58.3	53.6	55.9	51.4	56.1
Fukui	49.1	54.8	51.6	50.6	48.1	55.2	55.6	51.0	55.3	54.7	55.3	55.8	58.9
Yamanashi	62.5	71.3	77.2	75.8	64.6	70.8	67.3	59.7	66.7	68.4	62.0	66.3	70.0
Nagano	61.3	60.1	64.2	63.3	65.8	61.2	63.1	64.7	63.5	65.1	63.1	60.1	66.1
Gifu	57.8	56.0	57.3	58.6	59.9	60.4	57.2	53.0	58.3	55.2	57.1	55.1	62.1
Shizuoka	67.3	64.1	68.5	63.5	66.6	65.7	64.9	64.8	65.4	65.4	62.2	62.8	61.3
Aichi	62.0	62.7	63.9	62.0	61.2	61.9	61.3	61.8	62.0	62.1	58.9	61.6	60.0
Mie	58.8	51.9	56.1	55.0	54.6	54.7	54.8	60.9	54.5	53.7	55.1	51.2	57.8
Shiga	56.6	58.8	57.9	58.7	62.8	59.1	58.1	62.2	60.2	60.4	61.1	57.0	58.6
Kyoto	57.5	60.2	61.8	62.3	60.7	61.0	61.5	60.3	62.1	61.4	61.5	58.0	60.0
Osaka	62.6	62.2	62.1	63.1	61.1	60.8	59.6	61.2	59.4	57.8	57.3	56.2	57.4
Hyogo	58.3	57.6	59.2	57.7	58.2	57.8	59.3	58.6	56.5	60.0	57.6	57.5	55.6
Nara	67.1	61.5	61.0	61.0	61.9	60.7	60.3	59.0	59.1	60.5	54.3	59.0	58.2
Wakayama	52.6	62.2	59.3	56.9	61.9	60.4	56.7	62.5	63.4	58.9	61.2	56.5	60.4
Tottori	53.2	57.5	57.2	65.9	68.6	65.0	66.0	63.1	61.0	67.3	67.8	67.0	66.2
Shimane	53.8	63.7	71.9	71.9	69.6	66.5	63.5	74.1	65.5	64.5	61.9	61.5	64.5
Okayama	58.7	55.4	62.6	53.5	55.7	60.1	60.7	62.1	61.0	55.6	62.4	60.9	52.5
Hiroshima	59.1	59.0	62.2	60.6	61.7	61.2	59.6	60.5	58.6	61.2	57.4	59.8	57.2
Yamaguchi	60.1	63.5	64.6	62.5	61.5	63.5	62.4	62.2	64.5	62.2	60.9	61.3	67.2
Tokushima	50.7	50.8	56.2	59.0	52.5	49.2	58.1	55.7	61.5	62.7	56.6	54.7	57.7
Kagawa	60.3	61.0	59.4	64.1	56.4	62.2	54.8	58.4	59.2	61.5	58.5	60.6	64.2
Ehime	58.4	57.3	59.5	59.9	62.4	55.8	56.6	57.4	60.2	56.4	62.0	59.9	56.7
Kochi	68.0	70.6	63.1	63.9	63.0	67.8	68.3	70.8	66.7	57.7	68.7	56.3	74.0
Fukuoka	66.5	63.6	62.1	64.3	60.9	61.9	63.3	62.4	65.1	61.1	61.9	62.4	60.0
Saga	59.5	62.1	56.6	63.6	59.8	60.2	60.5	60.8	58.4	58.9	60.0	56.0	61.8
Nagasaki	59.6	62.7	59.7	61.8	57.3	55.4	55.0	57.1	55.8	55.7	57.4	54.3	54.5
Kumamoto	62.5	60.3	60.8	56.5	58.8	59.6	54.7	57.3	57.3	56.5	58.2	56.2	60.0
Oita	62.1	59.8	59.3	63.7	54.5	56.6	57.7	54.5	56.4	61.6	60.9	60.9	63.5
Miyazaki	60.3	67.7	61.6	59.6	63.7	60.8	65.2	64.8	61.8	59.5	63.8	62.5	62.1
Kagoshima	59.2	60.8	67.0	62.6	63.1	63.3	63.8	66.3	64.7	64.0	73.5	65.9	66.9
Okinawa	68.1	63.8	69.8	67.9	65.2	70.7	68.4	68.7	65.1	67.1	66.1	65.6	63.2

**Table 4:** The overall low birth weight rate and the term low birth weight rate per 1000 births in 2007–2019 for each prefecture and their APCs.

	Overall Low Birth Weight Rate		Term Low Birth Weight Rate	
Prefecture	Rate	APC	Rate	APC
Hokkaido	95.1	−0.41 (−0.81, −0.01)	57.7	−0.42 (−0.79, −0.04)
Aomori	92.5	−0.31 (−1.09, 0.47)	56.7	0.40 (−0.13, 0.93)
Iwate	94.4	0.86 (0.51, 1.21)	57.3	0.64 (0.02, 1.27)
Miyagi	94.1	0.32 (−0.10, 0.74)	55.6	0.31 (−0.19, 0.82)
Akita	99.9	0.42 (−0.07, 0.91)	61.4	1.17 (0.36, 1.98)
Yamagata	88.1	0.47 (−0.06, 1.01)	54.5	0.51 (−0.39, 1.43)
Fukushima	94.0	−0.03 (−0.62, 0.55)	60.0	0.29 (−0.47, 1.06)
Ibaraki	95.1	−0.04 (−0.39, 0.32)	60.9	0.22 (−0.12, 0.56)
Tochigi	104.1	0.16 (−0.21, 0.53)	65.2	0.59 (0.09, 1.08)
Gunma	95.1	0.03 (−0.39, 0.44)	61.0	−0.15 (−0.69, 0.39)
Saitama	94.4	−0.07 (−0.26, 0.12)	59.5	−0.18 (−0.41, 0.05)
Chiba	91.3	−0.17 (−0.43, 0.09)	57.6	−0.12 (−0.43, 0.20)
Tokyo	93.1	−0.38 (−0.53, −0.22)	59.5	−0.33 (−0.50, −0.15)
Kanagawa	95.4	−0.23 (−0.39, −0.07)	61.4	−0.52 (−0.81, −0.22)
Niigata	91.0	0.33 (−0.19, 0.85)	55.9	0.42 (−0.30, 1.14)
Toyama	89.8	−0.41 (−1.40, 0.59)	55.8	0.36 (−0.83, 1.56)
Ishikawa	87.8	0.67 (−0.06, 1.41)	54.2	0.70 (−0.30, 1.71)
Fukui	86.4	−0.21 (−0.82, 0.41)	53.4	1.04 (0.31, 1.79)
Yamanashi	104.1	−0.44 (−1.23, 0.37)	68.0	−0.54 (−1.77, 0.70)
Nagano	95.5	−0.39 (−0.81, 0.03)	63.1	0.25 (−0.28, 0.77)
Gifu	92.8	0.06 (−0.46, 0.58)	57.5	−0.02 (−0.74, 0.70)
Shizuoka	101.0	−0.42 (−0.57, −0.27)	64.9	−0.55 (−0.94, −0.16)
Aichi	97.3	−0.07 (−0.29, 0.15)	61.7	−0.32 (−0.58, −0.07)
Mie	91.3	0.21 (−0.24, 0.66)	55.3	−0.12 (−0.91, 0.69)
Shiga	94.1	−0.25 (−0.67, 0.17)	59.4	0.18 (−0.35, 0.71)
Kyoto	97.1	−0.49 (−0.64, −0.34)	60.6	0.03 (−0.39, 0.45)
Osaka	94.5	−0.70 (−0.92, −0.48)	60.2	−0.91 (−1.15, −0.67)
Hyogo	95.5	−0.43 (−0.66, −0.20)	58.0	−0.18 (−0.50, 0.14)
Nara	92.7	−0.63 (−1.15, −0.10)	60.4	−0.91 (−1.42, −0.40)
Wakayama	94.5	−0.25 (−0.86, 0.36)	59.4	0.36 (−0.50, 1.23)
Tottori	96.8	1.15 (0.57, 1.72)	63.4	1.41 (0.43, 2.41)
Shimane	101.9	0.26 (−0.82, 1.36)	65.6	−0.01 (−1.43, 1.42)
Okayama	89.1	−0.28 (−1.00, 0.44)	58.5	0.06 (−0.98, 1.12)
Hiroshima	96.2	−0.01 (−0.32, 0.29)	59.9	−0.29 (−0.69, 0.11)
Yamaguchi	96.9	−0.36 (−0.82, 0.11)	62.8	0.17 (−0.32, 0.67)
Tokushima	90.9	0.29 (−0.79, 1.39)	55.7	0.99 (−0.10, 2.11)
Kagawa	89.7	−0.06 (−0.68, 0.57)	60.0	0.11 (−0.66, 0.88)
Ehime	90.6	−0.14 (−0.68, 0.40)	58.7	−0.01 (−0.63, 0.62)
Kochi	105.7	−0.57 (−1.55, 0.43)	66.1	−0.24 (−1.57, 1.11)
Fukuoka	99.1	−0.59 (−0.79, −0.39)	62.8	−0.41 (−0.81, −0.01)
Saga	93.7	−0.21 (−0.75, 0.34)	59.9	−0.18 (−0.77, 0.42)
Nagasaki	91.7	−0.24 (−0.86, 0.38)	57.5	−0.95 (−1.44, −0.46)
Kumamoto	93.8	−0.77 (−1.27, −0.27)	58.4	−0.48 (−1.03, 0.08)
Oita	93.1	0.55 (−0.05, 1.16)	59.3	0.14 (−0.77, 1.06)
Miyazaki	103.1	−0.37 (−0.72, −0.02)	62.6	−0.04 (−0.69, 0.61)
Kagoshima	105.7	0.50 (−0.02, 1.03)	64.5	0.92 (0.24, 1.60)
Okinawa	112.2	−0.23 (−0.69, 0.24)	66.9	−0.32 (−0.86, 0.22)

APC, annual percent change.

**Table 5:** Median (Q1–Q3) for each characteristic in the prefectures used in the analysis.

Variable	Median (Q1–Q3)
Proportion of multiple births (%)	1.9 (1.8–2.1)
Proportion of live births from mothers aged under 20 years (%)	1.2 (1.0–1.4)
Proportion of live births from mothers aged 40 years or more (%)	4.7 (4.4–5.3)
Number of hospitals per 100,000 persons	7.1 (5.9–10.0)
Number of clinics per 100,000 persons	81.1 (72.3–89.6)
Number of hospitals with an obstetrics department per 100,000 persons;	1.3 (1.0–1.5)
Total population	1,642,589 (1,102,517–2,826,000)
Population density (population per hectare)	8.2 (6.2–12.3)
Taxable income per capita (1000 yen)	1287.1 (1141.3–1401.9)
Proportion of female high school graduates going to a higher educational institution	53.9 (49.3–57.6)
Proportion of live births from unemployed households	1.6 (1.3–2.1)
Proportion of obese women (BMI ≥ 25 kg/m^2^) (%) *	17.7 (16.0–19.8)
Proportion of lean women (BMI < 18.5 kg/m^2^) (%)	12.8 (11.7–13.9)

BMI, body mass index; * In Japan, BMI ≥ 25 kg/m^2^ is generally classified as obesity.

**Table 6:** Results of the regression analysis (N = 282).

Variable	Overall Low Birth Weight Rate		Term Low Birth Weight Rate	
	SPRC (95% CI)	*p*-Value	SPRC (95% CI)	*p*-Value
Year	−0.021	0.004	−0.029	0.001
	(−0.034, −0.008)		(−0.045, −0.012)	
Proportion of multiple births (%)	0.016	<0.001	0.011	<0.001
	(0.012, 0.021)		(0.005, 0.018)	
Proportion of live births from mothers aged under 20 years (%)	0.001	0.926	0.000	0.945
	(−0.010, 0.011)		(−0.012, 0.016)	
Proportion of live births from mothers aged 40 years or more (%)	0.010	0.087	0.026	<0.001
	(−0.001, 0.022)		(0.011, 0.040)	
Number of hospitals per 100,000 persons	−0.008	0.374	−0.002	0.862
	(−0.023, 0.008)		(−0.020, 0.017)	
Number of clinics per 100,000 persons	0.008	0.381	0.008	0.458
	(−0.009, 0.025)		(−0.012, 0.029)	
Number of hospitals with an obstetrics department per 100,000 persons;	−0.007	0.334	−0.015	0.114
	(−0.021, 0.007)		(−0.031, 0.003)	
Total population	0.007	0.714	−0.005	0.823
	(−0.026, 0.040)		(−0.044, 0.035)	
Population density (population per hectare)	−0.003	0.875	0.004	0.867
	(−0.036, 0.030)		(−0.036, 0.042)	
Taxable income per capita (1000 yen)	−0.016	0.303	−0.027	0.167
	(−0.045, 0.014)		(−0.061, 0.010)	
Proportion of female high school graduates going to a higher educational institution	−0.003	0.729	−0.002	0.881
	(−0.021, 0.014)		(−0.023, 0.020)	
Proportion of live births from unemployed households	0.005	0.466	−0.006	0.475
	(−0.008, 0.017)		(−0.021, 0.010)	
Proportion of obese women (BMI ≥ 25 kg/m^2^) (%) *	0.053	<0.001	0.071	<0.001
	(0.028, 0.079)		(0.039, 0.103)	
Proportion of lean women (BMI < 18.5 kg/m^2^) (%)	0.027	0.008	0.058	<0.001
	(0.008, 0.047)		(0.032, 0.082)	

SPRC, standardized partial regression coefficient; CI, confidence intervals; BMI, body mass index. * In Japan, BMI ≥ 25 kg/m^2^ is generally classified as obesity.

## Data Availability

The low birth weight data used in this study were obtained from the Ministry of Health, Labor, and Welfare in Japan. The results shown in this study were processed and analyzed by the authors, and are different from the statistics published by the Ministry of Health, Labor, and Welfare. In addition, the data on characteristics of the prefectures can be obtained from the websites of government statistics in Japan.
